# The Ergogenic Potential of Succinic Acid in Exercise Performance and Post-Exercise Recovery: A Systematic Review

**DOI:** 10.3390/nu18050870

**Published:** 2026-03-09

**Authors:** Karol Jędrejko, Oliver Catlin, Maciej Jędrejko, Bożena Muszyńska, Izabela Bat, Susan M. Kleiner, Dominika Granda, Andrzej Pokrywka, Ralf Jäger

**Affiliations:** 1 Department of Medicinal Plant and Mushroom Biotechnology, Faculty of Pharmacy, Jagiellonian University Medical College, 30-688 Krakow, Poland; maciej.jedrejko@proton.me (M.J.); bozena.muszynska@uj.edu.pl (B.M.);; 2 Banned Substances Control Group (BSCG), 11301 W. Olympic Blvd Suite 685, Los Angeles, CA 90064, USA; ocatlin@bscg.org; 3 High Performance Nutrition LLC, 9355 SE 47th Street, Mercer Island, WA 98040, USA; susan@drskleiner.com; 4 Department of Nutrition Physiology, Institute of Sport, National Research Institute, 01-982 Warsaw, Poland; dominika.granda@insp.pl; 5 Department of Biochemistry and Pharmacogenomics, Faculty of Pharmacy, Medical University of Warsaw, 02-097 Warsaw, Poland; andrzej.pokrywka@wum.edu.pl; 6 Increnovo LLC, 730 E Carlisle Ave, Whitefish Bay, WI 53217, USA

**Keywords:** succinate, endurance, ergogenic aid, energy booster, energotropic agent, acid-base balance, buffering agent, antioxidant capacity

## Abstract

**Background**: Succinic acid plays a central role in human energy metabolism as a key intermediate of the Krebs cycle that releases energy accumulated as guanosine triphosphate (GTP). Through its conversion via succinate dehydrogenase (SDH), succinate directly links the Krebs cycle to oxidative phosphorylation (OXPHOS), contributing to adenosine triphosphate (ATP) production. Exercise induces pronounced changes in succinate concentrations in skeletal muscle, blood, and saliva, with responses influenced by training status, exercise modality, and intensity. **Objective**: This systematic review evaluated the effects of succinate-containing supplements or sole-ingredient succinic acid supplementation on exercise performance and post-exercise recovery in healthy trained individuals. **Methods**: The review was conducted in accordance with PRISMA guidelines. PubMed/MEDLINE, Scopus, Web of Science, and Google Scholar were searched without date restrictions. Interventional studies assessing succinate-containing supplementation with outcomes related to exercise performance or recovery were included. Methodological quality was evaluated using the Cochrane Risk of Bias 2 tool. This study was registered in advance with the International Prospective Register of Systematic Reviews (**PROSPERO, CRD420251237042**). **Results**: Six studies involving 153 participants (mean age: 23 years) met the inclusion criteria. Five of the six included studies were rated as having a high risk of bias, while the only study judged to be at low risk of bias reported no beneficial effects on exercise performance outcomes. Supplementation protocols included daily doses of 300–2040 mg for up to 21 days and a single acute dose of 30 mg/kg, with most interventions administering succinate as part of multi-ingredient formulations rather than as an isolated compound. Three studies reported ergogenic effects in direct performance metrics, including improvements in maximal oxygen uptake, oxygen consumption, anaerobic threshold power, and total work performed. Two additional studies demonstrated favorable physiological adaptations indirectly relevant to exercise performance, including improved acid-base regulation, hematological markers related to oxygen transport, and antioxidant status, although validated performance outcomes were not assessed. Substantial heterogeneity and overall methodological limitations precluded meta-analysis. **Conclusions**: Current evidence suggests that succinate-containing supplements or sole-ingredient succinic acid supplementation may enhance direct performance outcomes such as aerobic performance, total workload, and indirect physiological markers, e.g., acid-base balance, hematological indicators and antioxidant capacity in healthy trained individuals. However, given that the majority of included studies were at high risk of bias and the only low-risk study reported no ergogenic effects, current evidence does not provide reliable support for performance-enhancing benefits of succinate supplementation. Interpretation is further limited by the predominant use of multi-ingredient formulations, making it difficult to isolate the effects of succinic acid. While biologically plausible mechanisms exist, well-controlled trials using isolated succinic acid are required before conclusions regarding efficacy can be drawn.

## 1. Introduction

Succinic acid, also known as butanedioic acid or amber acid, is a four-carbon dicarboxylic organic acid that plays a central role in human energy metabolism. Under physiological conditions, it exists predominantly in its ionized form, succinate dianion, and as part of several metabolic intermediates, including succinyl-CoA, succinate dehydrogenase (SDH), argininosuccinate, adenylosuccinate, and succinylcarnitine. Succinate and succinyl-CoA are key intermediates of the Krebs cycle, while SDH serves as a critical enzymatic link between the Krebs cycle and oxidative phosphorylation (OXPHOS). Through these pathways, succinic acid contributes to cellular respiration and the oxidation of carbohydrates and fatty acids for ATP production [1,2,3]. However, the influence of succinic acid supplementation on physical performance and post-exercise recovery may not be solely related to its role as a crucial metabolic substrate. Succinate also regulates energy homeostasis by activating succinate receptor-1 (SUCNR1) and modulating hypoxia-inducible factor (HIF) expression. Recent evidence further shows that succinate serves as a paracrine signaling molecule, driving muscle remodeling via SUCNR1 activation, and regulates post-exercise recovery and acid-base balance (pH). Meanwhile, the effect of succinate on HIF expression may correspond with increased hemoglobin (Hgb) and red blood cell (RBC) levels, improved tissue oxygenation, stimulation of vascular endothelial growth factor (VEGF) production, and enhanced angiogenesis in skeletal muscle [3,4].

Scientific evidence indicates that succinate concentrations in skeletal muscle and blood change markedly during physical exertion compared with resting conditions [5,6,7]. In addition, SDH activity has been shown to correlate with maximal oxygen uptake (VO_2_max), a key determinant of aerobic performance [8,9]. Together, these observations suggest that succinate availability and metabolism may be relevant to exercise performance and metabolic adaptation. Nevertheless, current international sports nutrition guidelines, including those issued by the International Olympic Committee (IOC) and the International Society of Sports Nutrition (ISSN), do not address succinic acid supplementation as a potential ergogenic aid [10,11]. Similarly, national frameworks such as those of the Australian Institute of Sport (AIS), which strongly influence global practice, do not include succinate in their supplementation recommendations [12]. To date, only one Krebs cycle intermediate, citric acid (or citrate), has gained recognition as an ergogenic aid, with sodium citrate commonly used as an alternative buffering agent to sodium bicarbonate [11,13,14].

In Western sports medicine, the absence of succinic acid from sport nutrition guidelines and commercial products likely reflects a perceived lack of robust interventional evidence rather than an absence of biological plausibility [15]. In contrast, succinate-based supplements have a long history of use in Eastern Europe and Central Asia. In countries such as Russia, Ukraine, and Kazakhstan, succinic acid is commonly included as a primary active ingredient in dietary supplements, typically in doses ranging from 100 to 340 mg per tablet or capsule, under various brand formulations [16,17,18,19]. In these regions, succinate is often described as an energotropic or recovery-supporting agent and is considered in modern sport nutrition and sports medicine reviews and used clinically as a membrane-protective compound, including in the management of exercise-induced anemia [20,21].

It should be noted that succinic acid is frequently incorporated into multi-ingredient formulations, complicating attribution of observed effects to succinate alone. Only a limited number of products contain succinic acid as a sole active ingredient, and systematic evaluation of its isolated effects remains scarce. Despite its clear physiological relevance and longstanding regional use, comprehensive syntheses of interventional studies examining succinic acid as an ergogenic aid are lacking. Therefore, the aim of this systematic review was to evaluate the available evidence on succinate-based supplement or succinic acid-based supplementation in healthy individuals, with a focus on its effects on exercise performance and post-exercise recovery and serve as a foundation for future research on its ergogenic potential.

## 2. Materials and Methods

### 2.1. Protocol and Registration

A systematic review of the literature was conducted following the Preferred Reporting Items for Systematic Review and Meta-Analysis (PRISMA) guidelines, maintaining transparency and methodological rigor throughout the processes of data selection, extraction, and synthesis [22]. The research question was structured according to the PICO acronym (Population, Intervention, Comparison, and Outcome). The Population investigated were healthy individuals; the Intervention was succinate-based supplements or succinic acid-based supplementation; the Comparison was placebo or baseline results; the Outcome was exercise performance parameters or post-exercise recovery indicators. This study was registered in advance with the International Prospective Register of Systematic Reviews (PROSPERO, CRD420251237042).

### 2.2. Search Strategy

Relevant articles were identified through an extensive electronic search of three online databases: PubMed/Medline, Scopus, Web of Science without any time frame restrictions. An initial systematic review of the literature was conducted in the period from 1 March 2025 to 26 June 2025.

The following search terms were used: ((succinic acid) OR (succinate) OR (amber acid) OR (amberen) OR (yantarin) OR (ЯнтарИн-Спорт [EN: Yantarin-Sport]) OR (янтарин [EN: amber])) AND ((exercise) OR (performance) OR (muscles) OR (physical) OR (endurance) OR (ergogenic) OR (oxygenation) OR (recovery) OR (hypoxia) AND (randomized controlled trials)).

Additionally, a manual search was performed using Google Scholar to identify other potentially eligible studies.

The search strategy incorporated terms related to randomized controlled trials (RCTs) to improve retrieval of controlled intervention studies; however, eligibility criteria were not restricted to RCTs and encompassed all interventional study designs. The detailed search strategies conducted are presented in Supplementary Materials, Table S1.

### 2.3. Inclusion and Exclusion Criteria

The inclusion criteria consisted of: (1) interventional study design; (2) studies involving healthy individuals of both genders, including athletes; (3) studies involving dietary supplement interventions; (4) articles written in English, Russian, or Ukrainian language; and (5) studies that included results associated with exercise/endurance performance or recovery.

The exclusion criteria were as follows: (1) in vitro studies; (2) animal studies; (3) observational or other non-interventional studies; (4) review articles; (5) studies involving non-healthy individuals; (6) studies involving medical treatment; and (7) studies that did not report outcomes related to exercise or endurance performance.

### 2.4. Study Selection and Data Extraction

Titles and abstracts of the identified studies were independently screened for eligibility by four co-authors (D.G., A.P., K.J., and M.J.). Subsequently, full-text articles were assessed in detail based on the predefined inclusion and exclusion criteria.

Any disagreements were discussed and resolved with the involvement of other co-authors (O.C., R.J., and S.M.K.). When essential information was missing from potentially eligible studies, the corresponding authors were contacted to obtain the necessary details.

The following data were extracted from the studies in terms of characteristics: name of the authors, study design, number and age of participants. Moreover, the information extracted in terms of methodology or results were: duration of the intervention, intervention design, dose and form of supplements, investigated parameters or measured outcomes, results, final outcome. Data regarding the safety assessment and the full composition of one capsule/tablet of the administered supplement were also collected.

### 2.5. Quality Assessment

The quality of the studies was independently assessed by two authors (K.J., and M.J.) using the Cochrane Collaboration’s Risk of Bias tool (RoB 2) [23]. Each study was evaluated based on several criteria, including random sequence generation, allocation concealment, blinding of participants and personnel, blinding of outcome assessment, incomplete outcome data, selective outcome reporting, and other potential sources of bias. Any disagreements were resolved through thorough re-reviewing until a consensus was reached.

Each study was assessed across five distinct domains: (1) bias arising from the randomization process; (2) bias due to deviations from intended interventions; (3) bias due to missing outcome data; (4) bias in measurement of the outcome; and (5) bias in selection of the reported result. The overall risk of bias was categorized as low, some concerns (unsure), and high.

## 3. Results

The initial multi-database search identified 4910 records: 998 from PubMed, 3744 from Scopus, and 168 from Web of Science. After removing 211 duplicate records, 4699 publications were screened by title and abstract. Of the 4699 records, 4488 were excluded based on title and abstract screening, primarily due to lacking results related to athletes, training or physical activity, relevance to medical treatment, analytical tests, in vitro tests, animal studies or review articles. The remaining 211 publications were selected for a full-text eligibility assessment.

**Figure 1:**
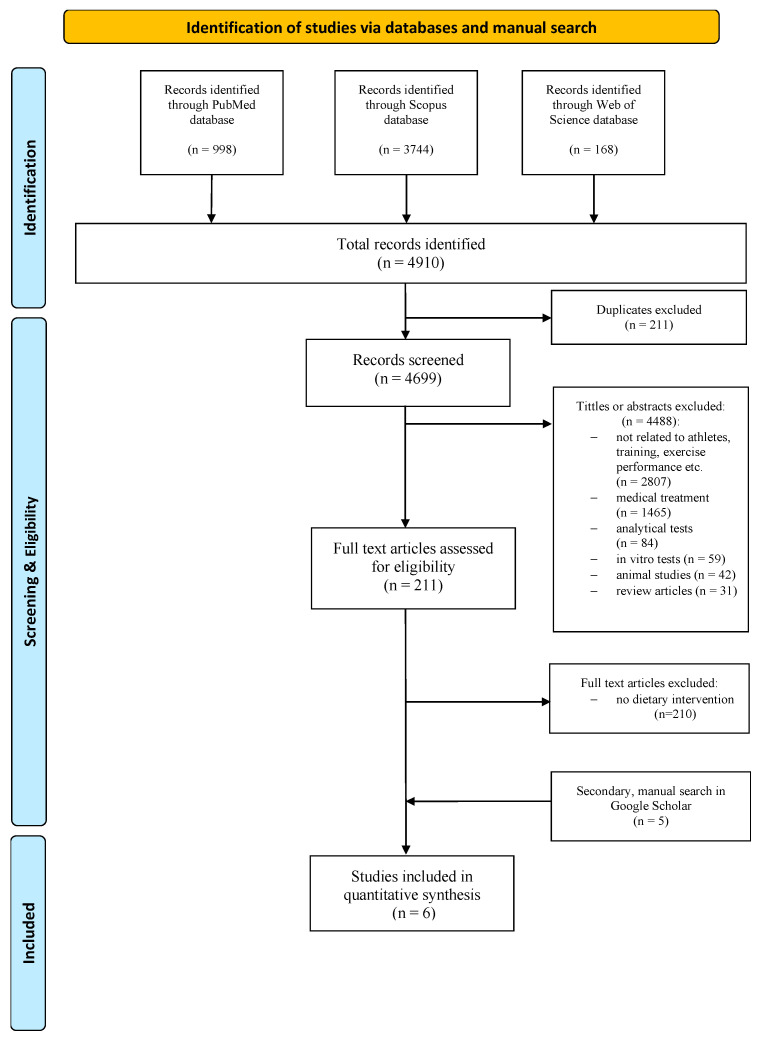
Literature review flow diagram of the selection process according to the Preferred Reporting Items for Systematic Reviews (PRISMA) Statement.

After applying the inclusion and exclusion criteria, 210 studies were excluded for not involving dietary supplement interventions in healthy individuals. Although the initial search yielded a high number of records, only one study met the predefined inclusion criteria, likely reflecting the limited research available on this specific topic. Therefore, an additional manual search was conducted using Google Scholar, which led to the identification of five articles meeting the inclusion criteria. In total, six studies (*n* = 6) were included in this systematic review. The selection of studies for this review was performed according to the PRISMA flow chart (Figure 1).

### 3.1. Description of Included Studies

The eligible studies were published between 2004 and 2019. Most studies were conducted in Eastern European countries, including four in Russia and one in Ukraine, while only one study was conducted in the United States. Across all studies, a total of 153 participants were included, covering 113 men and 18 women, with the caveat that in one study the sex of the 22 participants was not specified; with a mean age of 23 years (range 18–28). Supplementation periods averaged 21 days, with daily doses of succinate ranging from 300 to 2040 mg and exercise protocols varied across studies.

The first study, conducted by Brown et al., investigated the effects of a multi-ingredient dietary supplement containing a high dose of succinic acid (1000 mg per daily dose across six tablets) along with L-ornithine-L-aspartate, betaine, and several vitamins and minerals (in total of 52 different ingredients) in seven male cyclists. After 21 days of supplementation, the study found no ergogenic benefits. The supplement had no significant effect on cycling performance or post-exercise recovery, with no significant differences in key parameters such as the respiratory exchange ratio (RER), time to exhaustion (TTE), and lactate concentration [24].

The second study evaluated the effects of 21-day administration of multi-ingredient succinate-based supplement in 18 young female speed skaters classified as First-Class Athletes or Master of Sport candidates and did show improvement in performance related measurements. Athletes received either 300 mg of succinate-based supplement or a placebo. Participants completed two exercise tests: a submaximal cycle ergometer step test and, two weeks later, a maximal cycle ergometer step test. Blood samples were collected before and after exercise to evaluate acid-base status, including both metabolic (bicarbonate concentration) and respiratory (partial pressure of carbon dioxide) components of blood pH. Results indicated that succinate supplementation attenuated acid-base shifts during submaximal exercise compared to placebo. Under maximal load, it improved tolerance to acidosis and enabled athletes to complete a significantly greater workload. Performance improvements persisted for 2–3 h under submaximal conditions but were shorter during maximal exertion, with improvements lasting approximately 15 min before returning to baseline. Notably, athletes achieved greater workloads despite experiencing more pronounced acidosis [25].

Another study focused on hematological outcomes in 36 rowers and kayakers, 20 of whom received multi-ingredient succinate-based supplements (2040 mg/day of succinic acid formulated with glutamic acid, L-arginine, vitamin B6, and vitamin B2). The RBC count, Hgb levels, mean corpuscular volume/erythrocyte size (MCV), were assessed after 21 days of succinate supplementation. For comparison, homeostasis markers were also analyzed in 10 healthy, untrained individuals matched for age and gender. The results showed normalization of MCV, along with increases in Hgb levels and RBC count. These changes may indirectly enhance oxygen saturation in erythrocytes and improve oxygen transport to tissues [26].

Expanding on performance-related endpoints, a fourth study evaluated 30 highly trained athletes from a national track and field team. The study group received a multi-ingredient succinate-based supplement for 21 days (total of 2040 mg/day of succinic acid was used daily along with additional ingredients that played a role as cofactors). Exercise performance was assessed at the beginning and end of supplementation period using the Physical Work Capacity test at a heart rate of 170 bpm (PWC170). Additionally, changes in acid-base balance (pH) were analyzed. The study group showed a significant increase in PWC170 performance, from 16.44 to 20.52 W/kg, while the control group showed no ergogenic improvement. Blood pH in the placebo group tended to increase from whereas a significant decrease was observed in the study group, suggesting that succinate supplementation may help mitigate the development of metabolic acidosis during exercise. Antioxidant effects were also observed, including reduced lipid peroxidation, as indicated by decreased malondialdehyde (MDA) levels, and increased glutathione (GSH) accumulation in erythrocytes membranes. Succinate-based supplements were well tolerated, with no adverse effects reported over the 21-day period [27].

To compare different succinate-based formulations, a fifth study assessed the effects of a multi-ingredient succinate-based supplement versus a succinate-based drug, Armadin Long (emoxypine succinate; also known as Mexidol) in 40 young male weightlifters over 21 days. Both interventions contributed to improved antioxidant balance and normalized erythrocyte size, thereby enhancing hemoglobin saturation and oxygen transport to tissues. Both the succinate supplement and Armadin Long significantly reduced MDA levels compared to the placebo group, with a slightly greater reduction observed in the Armadin Long group. Additionally, both treatments increased GSH levels, with the succinate-based supplement group showing a slightly higher peak compared to the placebo [28].

The final study investigated the acute effects of a single dose of ammonium succinate (30 mg/kg body weight) taken 30 min prior to a maximal cycle ergometer step test. The participants were young elite athletes, including weightlifters, wrestlers, cyclists, and football players. The study focused on key cardiorespiratory parameters such as RER, oxygen consumption, respiration rate, pulmonary ventilation, anaerobic threshold, and VO_2_max [29]. During the maximal endurance test, acute intake of ammonium succinate enhanced oxygen utilization. VO_2_max increased by 9% (from 67 to 73 mL/kg/min), and RER rose by 4%, indicating increased carbon dioxide production relative to oxygen consumption. Oxygen consumption increased by 12.15%, while oxygen uptake efficiency improved, as evidenced by an 8.9% reduction in oxygen uptake costs. Ventilatory parameters improved by 22%, suggesting enhanced oxygen saturation in muscle tissues and reduced oxygen levels in exhaled air. Although the average TTE increased by 28 s (4%), this change was not statistically significant [29].

Among the six included studies, one investigation reported no evidence of ergogenic effects following administration of multi-ingredient succinate-based supplement [24]. The remaining five studies documented physiological changes potentially relevant to exercise performance, although the degree to which these changes were accompanied by direct improvements in objective performance outcomes varied across studies [25,26,27,28,29].

Two studies primarily reported favorable alterations in hematological parameters and antioxidant status, however a direct translation of these changes into measurable performance improvements was not consistently evident. Specifically, Gunina (2011) and Voitenko et al. (2019) observed increases in RBC count and Hgb concentration, normalization of erythrocyte volume indices, and reductions in markers of oxidative stress [26,28]. In addition, three studies reported modulation of acid-base balance during or following exercise, including improved buffering capacity against metabolic acidosis [25,27,28].

Direct and significant improvements in exercise capacity were demonstrated in three studies that investigated objective performance endpoints [25,27,29]. Maevsky et al. reported enhanced tolerance to high-intensity workloads, reflected by a 30% increase in total work performed; however, performance outcomes were inferred indirectly rather than assessed using standardized measures such as VO_2_max or TTE [25]. Gunina (2012) reported substantial increases in PWC170 values and reductions in post-exercise heart rate after 21 days of supplementation, indicating enhanced aerobic efficiency [27]. Tambovtseva et al. confirmed acute ergogenic effects, demonstrating meaningful increases in VO_2_max (+9%), oxygen consumption (+12%), and anaerobic threshold power (+17%), suggesting a rapid improvement in mitochondrial oxygen utilization following a single succinate dose [29].

Each of the studies was reviewed in depth, and the reported effects on exercise performance or post-exercise recovery are outlined in Table 1.

### 3.2. Succinic Acid Safety Assessment

Two of the six studies included in this systematic review assessed safety of short-term succinate supplementation (up to 21 days). No adverse effects were reported, and the intervention was well tolerated by participants [27,28].

### 3.3. Risk of Bias Assessment

**Figure 2:**
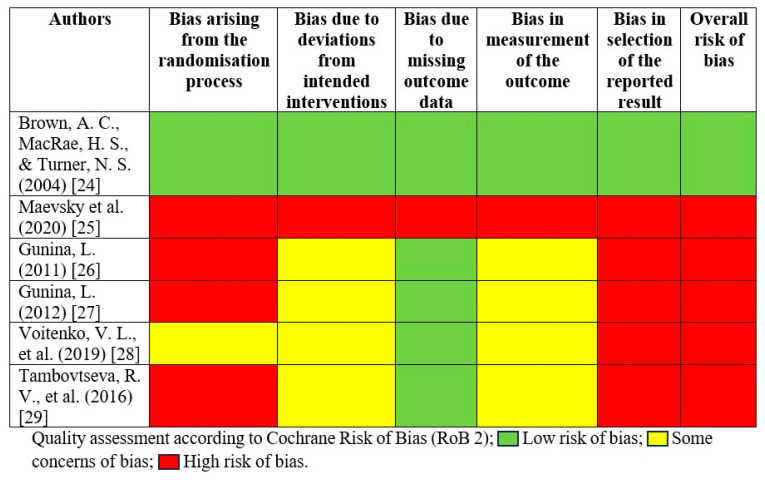
Quality assessment of studies included in the systematic review. Risk of Bias Heatmap [24,25,26,27,28,29].

According to the RoB2 assessment, only one of the included studies demonstrated a low overall risk of bias, meeting key methodological criteria such as appropriate randomization, blinding, complete outcome reporting, and transparent description of experimental procedures. The remaining five studies were assessed as having a high overall risk of bias, primarily due to insufficient reporting of randomization methods, unclear allocation concealment, and a lack of detail regarding blinding. In several cases, study protocols were not registered, and no predefined primary outcomes were reported, limiting the ability to evaluate selective reporting and increasing the likelihood of biased interpretation. While most studies presented full datasets with no participant dropouts, reducing concerns related to incomplete outcomes, this did not mitigate weaknesses in other critical domains of internal validity. The results of quality assessment are presented in Figure 2, with further details provided in the Supplementary Materials, Table S2.

Across the included literature, measurement bias was generally lower than other categories due to the use of objective physiological and laboratory parameters; however, the absence of documented blinding of outcome assessors and missing information about equipment calibration introduce uncertainty regarding measurement reliability. Only one study included detailed procedural safeguards, whereas others did not verify whether participants, researchers, or laboratory staff were blinded, potentially increasing performance or detection bias. As summarized in Figure 2, methodological inconsistencies across studies substantially limit confidence in reported effects and affect the comparability of outcomes. These findings underscore the need for future trials to employ robust randomized controlled designs with strict reporting transparency, pre-registration, predefined endpoints, and clearly described blinding and allocation procedures to enhance the credibility of conclusions regarding the ergogenic potential of succinic acid.

## 4. Discussion

This systematic review evaluated the effects of succinate-based supplementation on direct and indirect markers of exercise performance and recovery in trained individuals. Interpretation of the findings must consider that five of the six included studies were assessed as having a high risk of bias, while the only study judged to be at low risk of bias reported no performance-related benefits. In most studies, succinic acid was not administered as a single active ingredient but as part of multi-ingredient formulations. While co-formulation with metabolic cofactors has been proposed as a strategy to support bioenergetic pathways, this also limits the ability to attribute observed effects specifically to succinate. However, this results from the fact that in light of the original Russian research, the efficacy of succinic acid can be enhanced by employing formulation strategies that incorporate additional ingredients serving as cofactors, such as L-carnitine, vitamins B2, B3, B6, amino acids or organic acids (e.g., glutamic acid or fumaric acid) [27,30].

Several studies suggest that succinate-based supplements (or succinic acid) may be associated with improved lactate handling and oxidative utilization, potentially contributing to secondary ATP generation during periods of high metabolic demand [25,27,28]. In addition, favorable changes in antioxidant status and selected hematological parameters were reported in three studies, indicating potential systemic effects beyond performance-related outcomes [26,27,28].

The observed improvements in exercise capacity reported in three studies appear biologically plausible in light of succinate’s established metabolic functions, particularly its roles in OXPHOS and proton buffering under exercise-induced metabolic stress [25,27,29]. The described results concerning improvements in direct performance indicators, as well as indirect markers, should be approached with caution and reserve, since the majority of them derive from studies with serious methodological limitations.

The study by Brown et al., which was the only investigation assessed as having low risk of bias, reported no improvements in cycling performance or biochemical markers of recovery despite the use of a relatively high succinate dose. This finding stands in contrast to the positive outcomes reported in studies with higher risk of bias. As with most included studies, the intervention involved a multi-ingredient formulation; however, similar formulation complexity was also present in studies reporting positive findings. Therefore, the null result cannot be attributed solely to formulation characteristics and should be interpreted as the most methodologically robust estimate of effect currently available [24].

While several studies reported measurable physiological or performance-related outcomes following succinate-based supplementation, these findings were derived predominantly from studies with high risk of bias. When considering study quality, the only low-risk investigation did not demonstrate ergogenic effects. This underscores the importance of interpreting reported benefits within the context of methodological limitations rather than numerical consistency across studies. Although five of the six included investigations reported measurable physiological changes or performance-related outcomes following supplementation [25,26,27,28,29] these observations should be interpreted cautiously. In small, heterogeneous, and previously unregistered studies lacking pre-defined primary outcomes, such apparent consistency may reflect selective reporting, measurement bias, or publication bias within a regionally concentrated body of literature. Accordingly, the substantial high risk of bias, combined with heterogeneity in study design, dosing strategies, supplementation forms, and exercise protocols, limits definitive conclusions regarding a potential ergogenic role of succinic acid and underscores the need for standardized, high-quality randomized controlled trials.

### 4.1. Energotropic Effect of Succinic Acid and Its Role in Exercise Performance and Physiology

**Figure 3:**
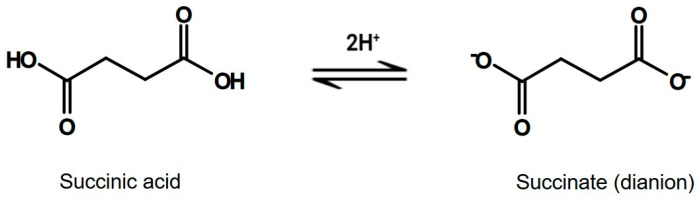
Chemical structure of succinic acid and succinate.

Succinic acid is a key intermediate in cellular energy metabolism, present in both intracellular and extracellular compartments. It plays a central role in the Krebs cycle and contributes directly to ATP production via OXPHOS. Beyond its metabolic function, succinate influences energy homeostasis through activation of SUCNR1 and modulation of HIF expression [3,4,31,32]. The chemical structures of succinic acid and succinate are shown in Figure 3.

During conversion of succinyl-CoA to succinate, energy (ΔG° = −33.5 kJ/mol) is conserved in the form of guanosine triphosphate (GTP), which is energetically equivalent to ATP. Subsequent oxidation of succinate to fumarate by SDH generates FADH_2_ and links the Krebs cycle to the respiratory chain. This reaction corresponds to the production of approximately 1.5 ATP equivalents, representing an energy release of ~46.3 kJ/mol [1,33].

**Figure 4:**
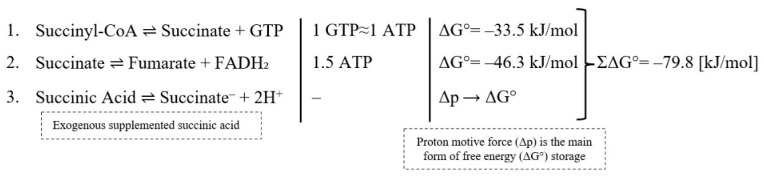
Free energy/energotropic effect of succinic acid and succinate.

Together, these steps yield an estimated ~79.8 kJ/mol, comparable to anaerobic glycolysis (ΔG° = −88 kJ/mol), emphasizing the substantial theoretical energotropic potential of succinic acid. Additionally, upon dissociation, succinic acid may also contribute to proton gradient formation and proton-motive force (Δp), which is the primary form of storing ΔG°, thereby indirectly supporting ATP synthesis, as shown in Figure 4. While the theoretical ΔG° associated with succinic acid exceeds that of creatine and associated phosphocreatine hydrolysis (1 ATP; ΔG° = −43.1 kJ/mol), these values reflect biochemical energy potential rather than direct comparative ergogenic efficacy.

Under conditions of metabolic stress such as hypoxia, ischemia, or intense exercise, enhanced succinate availability may support mitochondrial ATP production [1,33,34]. This supports a hypothesis that succinic acid supplementation could partially influence reliance on anaerobic glycolysis during high-intensity efforts such as swimming, middle-distance sprints (400–800 m) or weightlifting, lasting from approximately 20 s to 2 min; however, this concept remains theoretical and requires experimental validation. By delaying anaerobic glycolysis, succinic acid may help preserve the energy derived from this pathway (ΔG° = −88 kJ/mol) for later use during prolonged exercise.

Exercise induces measurable changes in succinate concentrations within muscle tissue, blood, and saliva [35,36,37,38]. Early studies demonstrated increased succinate accumulation following anaerobic exercise [39]. Subsequent human investigations have shown that succinate levels rise during the early stages of exertion and are influenced by training status, exercise modality, and intensity [40]. Higher post-exercise succinate concentrations have been reported following high-intensity exercise and among faster endurance athletes [5,41,42,43]. Elevated salivary succinate levels have also been observed following competition in female soccer players [44], and succinate concentrations have been shown to correlate with TTE in physically active men [45].

Huffman et al. reported increases in muscle succinate concentrations of up to 85% in well-trained individuals [46]. This was accompanied by elevated circulating succinate levels and increased hepato-splanchnic succinate flux during exercise in healthy young men [47]. Succinate has additionally been identified as a discriminatory metabolite associated with ketone body metabolism during exercise, suggesting potential shifts in substrate utilization [48]. Moreover, the formation of α-oxoglutarate, succinyl-CoA, and oxaloacetate from glutamate, valine, and isoleucine has been proposed as an anaplerotic pathway, which may further contribute to observed metabolic adaptations during exercise [48].

SDH activity, which is closely associated with aerobic capacity (VO_2_max), increases in response to endurance training. Elevated succinate levels and SDH activity therefore support the relevance of succinate in exercise adaptation [9,49,50]. Emerging evidence suggests that succinate may also act as a paracrine signaling molecule, promoting muscle remodeling through SUCNR1 activation and interaction with non-muscle cells involved in repair and adaptation [51,52].

**Figure 5:**
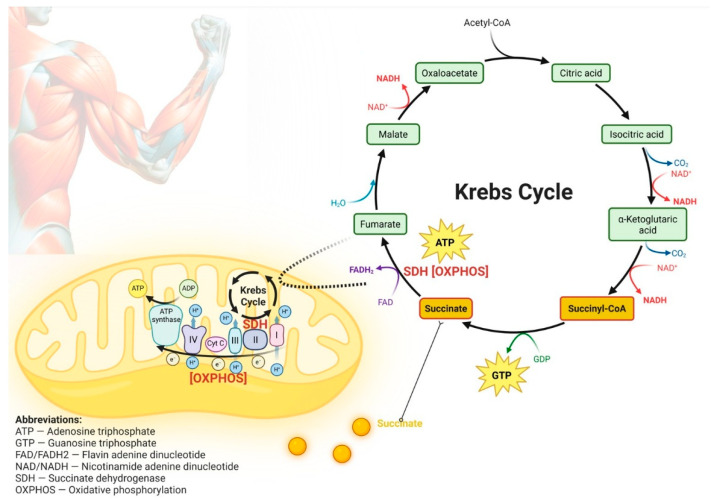
Energotropic effect of succinic acid [Created in BioRender. Jędrejko, K. (2025)] https://biorender.com/os3zyvf (accessed on 24 December 2025).

Recently, in a group of healthy adult individuals, supplementation with sole succinic acid has been shown to significantly increase ketone body levels in both urine and blood. Administration of succinic acid initiates a ketogenic effect without the involvement of additional individual factors such as diet or physical exercise. The ketogenic effect correlated with enhanced body composition in participants (reduction in body weight and BMI). Furthermore, the ketogenic effect (sustained over 2–4 weeks) may improve endurance performance in selected sports disciplines, by maximizing fat oxidation and providing a stable energy source, often leading to reduced fatigue during prolonged submaximal efforts. Succinic acid represents an alternative method (besides a ketogenic diet) for inducing elevated ketone body levels in the human body [53]. A summary of succinic acid’s energotropic effects is presented in Figure 5 [54].

### 4.2. Strengths and Limitations

To our knowledge, this is the first systematic review to examine the effects of succinate-based supplements or succinic acid-based supplementation on exercise performance and post-exercise recovery in healthy individuals. Key strengths include strict adherence to PRISMA guidelines, independent literature screening, and independent quality assessment of included studies, with disagreements resolved by senior authors.

The primary limitation of this review is the small number of eligible studies that qualified, five of which were assessed as having a high overall risk of bias. In addition, most studies originated from Russia or Ukraine, limiting external validity and potentially reflecting methodological differences. The limited regionality of the qualifying studies overlaps the limited geographical region where succinate is used as an ergogenic aid and that is unlikely to change without a systemic review. Other limitations include small and heterogeneous study populations, variability in supplementation protocols, and inconsistent outcome measures. An additional limitation of this review is the fact that the majority of the identified studies were located through manual searching on Google Scholar, without sufficient methodological detail to ensure reproducibility. Moreover, a limitation is that in five out of the six studies, multi-ingredient succinate-based supplements were used; succinic acid was not the sole active ingredient, and therefore causal inference regarding succinate itself is partially limited.

A meta-analysis was not feasible due to substantial heterogeneity and insufficient reporting of quantitative data, including effect sizes and measures of variance. Considering these limitations, the current body of evidence does not yet permit classification of succinic acid as an evidence-based ergogenic aid; however, the mechanistic plausibility and consistency of preliminary findings support further investigation.

### 4.3. Implications for Further Research

Although limited interventional studies have reported measurable physiological effects associated with succinic acid or succinate supplementation, its ergogenic potential in athletic populations remains unclear. The role of succinic acid in exercise performance and recovery is mechanistically supported but it is still insufficiently characterized and warrants further studies. Investigations using succinic acid alone should be considered to evaluate the isolated effects of succinic acid and elucidate further potential as a sports nutrition ingredient.

Future trials should employ larger, well-controlled designs to define optimal dosing strategies and evaluate efficacy across diverse athletic populations. Studies should include both male and female participants, with sex-specific analyses, given evidence that biological sex may influence the pharmacokinetics and metabolism of bioactive compounds [55]. Consistent use of well-recognized markers of exercise performance should be included.

Preliminary findings indicate potential effects on Hgb concentration and RBC count [26]. This may be relevant to prevent exercise-induced anemia (sports anemia), a condition known to impair endurance, recovery, and overall sports performance [56,57]. However, these observations remain speculative and require targeted investigation. The current evidence base is predominantly derived from male participants, which limits the generalizability of findings to females, and direct interventional evidence in iron-deficient or anemic populations is lacking.

Further research should also examine sole ingredient–succinic acid supplementation across different training modalities and sports disciplines, including endurance (e.g., cycling, running, swimming), resistance (e.g., weightlifting including deadlifts, squats, bench press), high-intensity (e.g., rowing, sprinting), and mixed exercise formats such as soccer, football, and basketball. Comparative studies against established ergogenic aids such as creatine, citrate or beta-alanine would provide additional context regarding relative efficacy.

Finally, extended human trials should include a thorough examination of pharmacokinetic parameters, such as the bioavailability of succinic acid after oral administration, distribution in body compartments (into various tissues), biotransformation (metabolism), half-life, and excretion route of succinic acid from the body (including elimination time).

The safety of succinic acid supplementation should also be assessed in terms of possible adverse effects in athletes under different dosing scenarios (maximum single dose, maximum daily dose, maintenance dose) and at different periods of supplementation, i.e., acute, short-term and long-term intervention.

## 5. Conclusions

The current evidence base evaluating succinate-based supplementation in exercise contexts is limited and characterized by substantial methodological concerns. Five of the six included studies were assessed as having a high risk of bias, and the only study judged to be at low risk of bias reported no ergogenic effects on exercise performance outcomes. Furthermore, in most studies, succinic acid was administered as part of multi-ingredient formulations, limiting causal attribution to succinate itself. Among the studies with high risk of bias, findings suggest that succinate-based supplementation may enhance direct performance metrics such as aerobic performance (VO_2_max, VO_2_) and total workload, as well as indirect physiological markers including acid-base balance, hematological parameters, and antioxidant capacity in healthy trained individuals. However, these findings are not supported by the only low-risk study and therefore do not provide reliable evidence of efficacy. While the central role of succinate in mitochondrial metabolism provides mechanistic plausibility for potential physiological effects relevant to performance and recovery, current clinical evidence remains insufficient to support ergogenic claims. Accordingly, well-designed randomized controlled trials using isolated succinic acid are required to determine whether mechanistic potential translates into meaningful performance outcomes.

## Figures and Tables

**Table 1:** Summary of studies considering supplementation of succinic acid or succinate-based supplements.

Reference	Study Design	Participant Characteristic	Mean Age, Years (±SD)	Duration of Supplementation	Intervention	Dose	Investigated Parameters	Outcomes Measured			Results	Outcome	Safety Assessment	Full Composition/Concomitant Ingredients per Single Dose
								**Baseline CMG**	**Main Group**	**Placebo**				
Brown et al. (2004) [24]	Double-blind controlled placebo	Well-trained male cyclist *n* = 7 M = 7 F = 0	28.6 ± 2.4	21 days supplementation during exercise tests; then 7 days washout period; another 21 days supplementation	Multi-ingredient dietary supplement with succinate as an main active ingredient **Placebo group**	1000 mg/day of succinic acid daily dose of 6 tablets per day; 3 tablets twice daily–3 tablets 1 h before workout and 3 tablets 1-h after workout	Cycle ergometer RER	N/D	0.96	0.95	No significant differences in RER	No ergogenic effect; no improvement in cycling performance and post-exercise speed recovery	No information	1 tablet ≈ 500 mg include: 166 mg succinic acid 80 mg malate 80 mg inosine 30 mg L-ornithine-L-aspartate 15 mg L-carnitine 15 mg trimethylglycine (betaine) vitamins and minerals (in total of 52 different ingredients)
							TTE (min)	N/D	105 ± 18	113 ± 11	No significant differences in TTE			
							Lactate	N/D	2.7–3.8	2.7–3.4	No significant differences in lactate concentration			
Maevsky et al. (2020) [25]	Double-blind controlled placebo	Speed skaters; First-Class Athletes or Master of Sport *n* = 18 M = 0 F = 18 9 administered Signalom	19–21 years	21 days	Multi-ingredient dietary supplement with succinate as an main active ingredient (Signalom) **Placebo group**	300 mg/day of succinate (ammonium succinate + succinic acid) daily dose of 2 capsules intake 20 min prior to effort	Submaximal cycle ergometer step test (3 min cycle on 300 W output)	N/D	~420	~340	Increase in total work by 30%	Ergogenic effect Correction of metabolic acidosis in response to physical effort	No information	1 capsule Signalom ≈ 300 mg include: 150 mg succinate (135 mg ammonium succinate + 15 mg succinic acid) 36 mg sodium fumarate 36 mg L-carnitine fumarate 30 mg sodium pyruvate 30 mg ascorbic acid 9 mg ammonium phosphate 9 mg sodium bicarbonate
							Acid-base balance (pH) in blood	~7.4	7.4 to 7.3	7.4 to 7.2	Normalization of pH			
Gunina (2011) [26]	Double-blind controlled placebo	Rowers, kayakers *n* = 36 M = 36 F = 0 20 administered YantarIn-Sport	21.3 ± 2.4	21 days	Multi-ingredient dietary supplement with succinate as an main active ingredient (YantarIn-Sport) **Placebo group**	2040 mg/day of succinic acid daily dose of 6 capsules; 2 cap30 min3 times daily–2 capsules in the morning; 2 capsules 30 min before workout; 2 capsules 30-min after workout	RBC	N/D	5.47 ± 0.14	4.76 ± 0.22	Increase RBC	Ergogenic effect has not been investigated Normalization of RBC size and MCV in response to physical effort Antioxidant effect	No information	1 capsule YantarIn–Sport ≈ 1100 mg include: 340 mg succinic acid 500 mg glutamic acid 200 mg L-arginine 40 mg vitamin B6 20 mg vitamin B2
							Hgb	N/D	159.7 ± 6.5	141.8 ± 7.5	Increase Hgb			
							MCV	N/D	74.7 ± 3.1	88.5 ± 4.8	Normalization of MCV			
							MDA	N/D	3.98 ± 0.06	4.61 ± 0.15	Decrease MDA			
							GSH	N/D	3.12 ± 0.09	2.34 ± 0.12	Increase GSH			
Gunina (2012) [27]	Double-blind, placebo controlled	Track and field athletes–members of the national team *n* = 30 M = 30 F = 0 15 administered YantarIn-Sport	N/D	21 days	Multi-ingredient dietary supplement with succinate as an main active ingredient (YantarIn-Sport) **Placebo group**	2040 mg/day of succinic acid daily dose of 6 capsules; 2 cap30 min3 times daily–2 capsules in the morning; 2 capsules 30 min before workout; 2 capsules 30-min after workout	Cycle ergometer PWC170 test Aerobic power [W/kg]	N/D	20.52 ± 0.29	16.44 ± 0.19	Increase in total work	Ergogenic effect Normalization of HR Correction of metabolic acidosis in response to physical effort Antioxidant effect	Safety assessment: no reported adverse effects; supplementation was well tolerated	1 capsule YantarIn–Sport ≈ 1100 mg include: 340 mg succinic acid 500 mg glutamic acid 200 mg L-arginine 40 mg vitamin B6 20 mg vitamin B2
							HR	N/D	135.5 ± 4.8	161.6 ± 7.9	Decrease HR after effort			
							Acid-base balance (pH) in blood	~7.4	7.41 ± 0.02 to 7.46 ± 0.02	7.42 ± 0.02 to 7.35 ± 0.01	Normalization of pH			
							MDA	N/D	5.61 ± 0.53	7.56 ± 0.45	Decrease MDA			
							GSH	N/D	3.04 ± 0.13	2.39 ± 0.08	Increase GSH			
Voitenko et al. (2019) [28]	Double-blind, placebo controlled	Young weightlifters; Masters of Spors *n* = 40 M = 40 F = 0 15 administered Armadin Long 13 administered YantarIn-Sport 12 administered Placebo	18–23	21 days	Multi-ingredient dietary supplement with succinate as an main active ingredient (YantarIn–Sport) Armadin Long **Placebo group**	2040 mg/day of succinic acid daily dose of 6 capsules; 2 capsules 3 times daily–2 capsules at morning; 2 capsules 30 min before workout; 2 capsules 30 min after workout 1800 mg/day of Armadin Long; two tablets 3 times daily	MCV	–	78.04 ± 1.86 to 80.25 ± 2.20	77.82 ± 1.6 to 88.43 ± 2.1	Similar results for Armadin Long and YantarIn–Sport	Ergogenic effect has not been investigated Similar results for Armadin Long and YantarIn–Sport Normalization of erythrocytes size and MCV in response to physical effort Correction of metabolic acidosis in response to physical effort Antioxidant effect	Safety assessment: no reported adverse effects; supplementation was well tolerated	1 capsule YantarIn–Sport ≈ 1100 mg include: 340 mg succinic acid 500 mg glutamic acid 200 mg L-arginine 40 mg vitamin B6 20 mg vitamin B2 1 tablet Armadin Long include 300 mg emoxypine succinate
							Acid-base balance (pH) in blood	~7.4	7.37 ± 0.02 to 7.38 ± 0.03	7.38 ± 0.02 to 7.28 ± 0.01	Normalization of pH			
							MDA	N/D	5.18 ± 0.05 to 6.04 ± 0.05	5.12 ± 0.04 to 7.21 ± 0.05	Decrease MDA vs. placebo group (slightly more reduction for Armadin Long)			
							GSH	N/D	2.32 ± 0.06 to 2.50 ± 0.05	2.21 ± 0.09 to 1.45 ± 0.05	Increase GSH vs. placebo group (slightly more peak for YantarIn–Sport)			
Tambovtseva et al. (2016) [29]	Comparative study	Elite athletes: weightlifters, wrestlers, cyclists and football players *n* = 22 Sex not specified	19–21	Acute intake	Ammonium succinate **Without placebo group**	30 mg/kg/b.w.	Maximal cycle ergometer step test RER	1.01 ± 0.02	1.05 ± 0.02	N/D	Improved tolerance to exercise RER increase by 4%	Ergogenic effect Improved oxygen saturation	No information	
							VO_2_max	67 ± 2	73 ± 2	N/D	VО_2_max increase by 9% in compare to baseline result			
							VO_2_	5.20 ± 0.21	4.74 ± 0.13	N/D	VO_2_ decreased by 8.9% compared to baseline result			
							Oxygen consumption [L/min]	2.14 ± 0.09	2.4 ± 0.09	N/D	Oxygen consumption increased by 12.1% compared to baseline result			
							Power on anaerobic threshold [W]	165.8 ± 8.01	194.8 + 10	N/D	Power on anaerobic threshold increased by 17.5% compared to baseline result			
							Relative power of anaerobic threshold [W/kg]	2.17 ± 0.11	2.52 ± 0.16	N/D	Relative power of anaerobic threshold increased by 16.1% compared to baseline result			

Abbreviations: M: males; F: females; GSH: glutathione; Hgb: hemoglobin; HR: heart rate; MCV: mean corpuscular volume; MDA: malondialdehyde; N/D: not determined; PWC170: physical working capacity; RBC: red blood cells count; TTE: time to exhaustion; VO_2_: oxygen uptake; VO_2max_: maximal oxygen uptake.

## Data Availability

The original contributions presented in this study are included in the article/Supplementary Materials. Further inquiries can be directed to the corresponding authors.

## Supplementary Materials

The following supporting information can be downloaded at: https://www.mdpi.com/article/10.3390/nu18050870/s1, Table S1: Search strategy, Table S2: Rob.

## Abbreviations

The following abbreviations are used in this manuscript:

GTP	Guanosine triphosphate
SDH	Succinate dehydrogenase
OXPHOS	Oxidative phosphorylation
ATP	Adenosine triphosphate
RoB 2	Cochrane Risk of Bias 2
VO_2_max	Maximal oxygen uptake
VO_2_	Oxygen uptake
IOC	International Olympic Committee
ISSN	International Society of Sports Nutrition
AIS	Australian Institute of Sport
RER	Respiratory exchange ratio
TTE	Time to exhaustion
RBC	Red blood cell count
Hgb	Hemoglobin
MCV	Mean corpuscular volume/erythrocyte size
PWC170	Physical Work Capacity test at a heart rate of 170 bpm
HR	Heart rate
MDA	Malondialdehyde
GSH	Glutathione
SUCNR1	Succinate receptor-1
HIFs	Hypoxia-inducible factors
