# Supplementary material for: The Ergogenic Potential of Succinic Acid in Exercise Performance and Post-Exercise Recovery: A Systematic Review
Source: Nutrients. 2026 Mar 9;18(5):870. doi: 10.3390/nu18050870 (PMC12986710; doi:10.3390/nu18050870)
Supplement: Supplementary file 1 [file nutrients-18-00870-s001.zip › Supplementary Table S1 Search strategy.pdf]

Supplementary Table S1. Search strategy

Databases	Search strategy	Limits	Results
PubMed	#1 ((succinic acid) OR (succinate) OR (amber acid) OR (amberen) OR (yantarin) OR (ЯнтарИн-Спорт) OR (янтарин))<br>#2 ((exercise) OR (performance) OR (muscles) OR (physical) OR (endurance) OR (ergogenic) OR (oxygenation) OR (recovery) OR (hypoxia))<br>#1 AND #2	Clinical Trial, Randomized Controlled Trial, Humans	998
Scopus	TITLE-ABS-KEY (((“succinic acid” OR “succinate” OR “amber acid” OR “amberen” OR “yantarin” OR “ЯнтарИн-Спорт” OR “янтарин”) AND (“exercise” OR “performance” OR “muscles” OR “physical” OR “endurance” OR “ergogenic” OR “oxygenation” OR “recovery” OR “hypoxia”)	**Limited to:**<br>Controlled study, Clinical trial<br>**Excluded:**<br>Nonhuman<br>Animal experiment	3744
Web of Science	(((“succinic acid” OR “succinate” OR “amber acid” OR “amberen” OR “yantarin” OR “ЯнтарИн-Спорт” OR “янтарин”) AND (“exercise” OR “performance” OR “muscles” OR “physical” OR “endurance” OR “ergogenic” OR “oxygenation” OR “recovery” OR “hypoxia”) AND (“randomized controlled trials”)))		168
