# Supplementary material for: The Ergogenic Potential of Succinic Acid in Exercise Performance and Post-Exercise Recovery: A Systematic Review
Source: Nutrients. 2026 Mar 9;18(5):870. doi: 10.3390/nu18050870 (PMC12986710; doi:10.3390/nu18050870)
Supplement: Supplementary file 1 [file nutrients-18-00870-s001.zip › Supplementary Table S2 RoB.pdf]

„Tricarboxylic-Acid-Cycle Intermediates and Cycle Endurance Capacity” (Brown et al., 2004)

Domain	Risk of Bias	Details
**1. Bias arising from the randomization process**	🟢 Low	The present study was conducted in accordance with the stipulated principles of a randomised, double-blind, crossover design. The sequence in which participants received either succinate or placebo was determined by a randomisation process. Despite the absence of specifications regarding the method of sequence generation (e.g. random number table or software), the description indicates a plausible random allocation. There is no indication of baseline imbalances between the groups, and the crossover design inherently helps to control for between-subject variability.
**2. Bias due to deviations from intended interventions**	🟢 Low	Both the participants and the researchers were unaware of the intervention. Interventions (succinate or placebo) were administered under the supervision of a qualified medical professional, and there is no indication of non-adherence or deviation from the stipulated protocol. The crossover nature of the trial is a further control for individual differences. The allocation was concealed and blinding was maintained, which minimises the likelihood of performance bias.
**3. Bias due to missing outcome data**	🟢 Low	The authors explicitly stated that all recruited participants completed the study. There is no indication of dropouts or exclusions from analysis. All participants were included in the analysis of both conditions, and the crossover design allowed full within-subject comparisons. Therefore, risk due to missing data is minimal.
**4. Bias in measurement of the outcome**	🟢 Low	The outcomes included objective physiological parameters, such as VO_2max, time to exhaustion, and lactate thresholds, measured using standardised equipment and procedures. The outcome assessors were unaware of the condition. The use of validated measurement methods, along with the blinding of personnel,
**5. Bias in selection of the reported result**	🟢 Low	The study reported all outcomes described in the methods section, including both primary (VO_2max, performance time) and secondary (blood markers) endpoints. There is no evident indication of selective reporting or unexplained omissions. It is noteworthy that the protocol had not been pre-registered (a common occurrence in 2004), yet all analyses exhibited signs of pre-planning. Furthermore, the statistical reporting is characterized by transparency and completeness.

Final score:

🟢 **Low risk of bias -** well-conducted randomized trial with blinding and transparent reporting of results.

„Effect of Sodium Succinate on Oxidative Stress in Erythrocytes during Physical Loading” (Gunina 2011)

Domain	Risk of Bias	Details

**1. Bias arising from the randomization process**	🔴 High	The study does not provide any information regarding the random assignment of participants to groups. The article makes mention of the 'splitting' of participants; however, it does not provide any indication whether randomisation was employed, or the method by which it was carried out. Furthermore, no mechanism has been provided for concealing the allocation.
**2. Bias due to deviations from intended interventions**	🟡 Some concerns	It is important to note that the study's authors did not provide any information on how participants or investigators were blinded. Additionally, the appearance and taste of the supplement and placebo were not described. Moreover, the study revealed a paucity of data pertaining to the monitoring of adherence to the supplementation protocol, which is an issue that should be addressed.
**3. Bias due to missing outcome data**	🟢 Low	The results of both groups of participants are shown in detail in the table. As the authors reported no loss of observations or exclusions, the risk of measurement bias is minimised.
**4. Bias in measurement of the outcome**	🟡 Some concerns	The analysed parameters (morphology, erythrocytes and enzyme activity) were measured by laboratory methods. Nevertheless, the study authors did not indicate whether the laboratories were blinded to the intervention group.
**5. Bias in selection of the reported result**	🔴 High	The article does not mention the registration of the study protocol. The results were presented selectively, focusing on the most significant hematological and enzymatic changes. Also there is no information about planning endopoints.

Final score:

🔴 **High risk of bias** – the main reasons are inadequate randomisation, a lack of overt blinding, and the fact that the study was not registered. There is also a potential risk of selective reporting of results.

„A validity of the use of compositions on the basis of succinic acid in higher achievements sports" (Gunina, et al. 2012)

Domain	Risk of Bias	Details
**1. Bias arising from the randomization process**	🔴 High	The authors point out that the groups are homogeneous in terms of age, gender and training experience, but there is no information about the random allocation of participants or the hidden allocation sequence. Furthermore, the study lacks a description of the randomisation process and allocation generation scheme.
**2. Bias due to deviations from intended interventions**	🟡 Some concerns	We do not have any information about whether the present study was blinded. The authors also did not describe the appearance and taste of the capsules containing the test ingredient or placebo. Although there is a risk of deviation due to lack of masking, there is nevertheless no evidence of a real failure to follow the protocol.
**3. Bias due to missing outcome data**	🟢 Low	The size of the pre- and post-intervention groups is identical, and the results presented in the tables are for all study participants. There is no information on exclusions or incomplete observations..
**4. Bias in measurement of the outcome**	🟡 Some concerns	VO_2 max, PWC 170 and biochemical parameters were measured in the laboratory, but there is no information on whether those responsible for these processes were blinded. Additionally, it was not stated whether the measurement equipment was independently calibrated.
**5. Bias in selection of the reported result**	🔴 High	The protocol was not registered. The results were selectively presented as changes in haematological parameters and PWC170, but the authors did not state whether other endpoints had been planned.

Final score:

🔴 **High risk of bias** – this study was potentially controlled, but there is insufficient evidence of randomisation, and important information was lacking, e.g. regarding blinding and registration of the entire protocol.

„Effects of Ammonium Succinate on Aerobic Performance of Athletes” (Tambovtseva, et al. 2016)

Domain	Risk of Bias	Details
**1. Bias arising from the randomization process**	🔴 High	The study does not detail the randomisation procedure or allocation masking. While the authors state that the groups were 'homogeneous in both age and training', they do not confirm the use of random assignment. Furthermore, no information is provided regarding the mechanism for concealing the randomisation sequence.
**2. Bias due to deviations from intended interventions**	🟡 Some concerns	The article does not provide any information on blinding in relation to the intervention and placebo administration, whether for participants or investigators. Furthermore, the authors did not indicate whether adherence to the protocol was monitored during the study.
**3. Bias due to missing outcome data**	🟢 Low	The authors noted no significant data gaps, and results were presented for all study participants. The article does not report on exclusions or lost observations either.
**4. Bias in measurement of the outcome**	🟡 Some concerns	The study reported the results of the performance tests (VO2max measurement, running test), but did not report whether the individuals responsible for measuring the results were blinded to group assignment. Additionally, the authors did not provide information on the standardisation and calibration of the measuring devices.
**5. Bias in selection of the reported result**	🔴 High	The article only presents selected results (e.g. running time and VO_2 max value) and does not refer to the previously recorded protocol or clinical trial registration. This raises concerns that the authors may have selected only statistically significant results that were beneficial to the study.

Final score:

🔴 **High risk of bias** – the study is missing crucial information. Specifically, it does not provide details on randomisation, nor does it include any data on blinding or registration protocol. This could mean that the results may have been reported selectively.

„Succinic acid-based products as safe and effective factors supporting homeostasis parameters during physical loads" (Voitenko et al., 2019)

Domain	Risk of Bias	Details
**1. Bias arising from the randomization process**	🟡 Some concerns	Despite the authors confirming that the study and samples were randomised, there was no comprehensive account of the sequencing mechanism (e.g. the use of a random number generator) or of the allocation concealment procedures. Information on whether randomisation of participants was carried out independently was also limited.
**2. Bias due to deviations from intended interventions**	🟡 Some concerns	The study was declared to be double-blind, but no details were provided. The authors did not indicate who was blinded or whether the supplement and placebo capsules were visually and taste-identical. The process by which the effectiveness of blinding was verified was not mentioned either.
**3. Bias due to missing outcome data**	🟢 Low	The results of both groups of participants are shown in detail in the table. As the authors reported no loss of observations or exclusions, the risk of measurement bias is minimised.
**4. Bias in measurement of the outcome**	🟡 Some concerns	Despite the study using laboratory methods and conducting stress tests, there is no information about the blinding of the analysts who analysed the samples. Furthermore, the authors did not provide information on the calibration of the apparatus used or the procedure for minimising the impact of the research team's expectations.
**5. Bias in selection of the reported result**	🔴 High	There is no recorded protocol or predefined endpoints. The authors present the results of many parameters, but it is unclear whether only statistically significant results were chosen. The authors present numerous parameters, but it is unclear whether only statistically significant results were selected.

Final score:

🔴 **High risk of bias** – although the study has a declared double-blinding and randomization, it lacks important methodological details such as allocation or registration of the study protocol.

„Formulation and dosage forms for enhancing performance or recovery from stress" (Mayevsky et al., 2020)

Domain	Risk of Bias	Details
**1. Bias arising from the randomization process**	🔴 High	The study does not detail the randomisation procedure or allocation masking.
**2. Bias due to deviations from intended interventions**	🔴 High	No mention of blinding or adherence.
**3. Bias due to missing outcome data**	🔴 High	No data on outcomes or participant flow.
**4. Bias in measurement of the outcome**	🔴 High	No measurable clinical outcomes described.
**5. Bias in selection of the reported result**	🔴 High	No pre-specified outcomes or results presentent

Final score:

🔴 **High risk of bias** – the referenced document provides a technical description of formulations containing succinic acid for enhancing physical performance or recovery. Although it includes conceptual claims and general observations, the document:

- Does not present a randomized controlled trial (RCT)
- Lacks any description of study design, such as randomization procedures, group allocation, or blinding
- Does not provide outcome data, participant numbers, or statistical analysis
- Does not qualify as a primary clinical study under the standards for systematic review or evidence grading
